# Service usage and vascular complications in young adults with type 1 diabetes

**DOI:** 10.1186/1472-6823-14-39

**Published:** 2014-05-09

**Authors:** Steven James, Lin Perry, Robyn Gallagher, Julia Lowe, Janet Dunbabin, Patrick McElduff, Shamasunder Acharya, Katharine Steinbeck

**Affiliations:** 1 Huntsville District Memorial Hospital, 100 Frank Miller Drive, Huntsville, Ontario P1H 1H7, Canada; 2 University of Technology, Sydney, 15 Broadway, Ultimo, New South Wales 2007, Australia; 3 Sunnybrook Health Sciences Centre, 2075 Bayview Avenue, Toronto, Ontario M4N 3M5, Canada; 4 University of Newcastle, University Drive, Callaghan, Newcastle, New South Wales 2308, Australia; 5 Hunter New England Local Health District, New Lambton Heights, New South Wales 2305, Australia; 6 University of Sydney, Darlington, Sydney, New South Wales 2008, Australia

**Keywords:** ‘Service usage’, Health services, ‘Vascular complications’, ‘Prevalence’, ‘Prediction’, ‘Retinopathy’, ‘Nephropathy’, ‘Hypertension’, ‘Blood pressure’, ‘Young adults’, ‘Type 1 diabetes’

## Abstract

**Background:**

Few studies have examined young adults with type 1 diabetes use of health services and the development of vascular complications. As part of the Youth Outreach for Diabetes (YOuR-Diabetes) project, this study identified health service usage, the prevalence and factors predictive of development of vascular complications (hypertension, retinopathy and nephropathy) in a cohort of young adults (aged 16–30 years) with type 1 diabetes in Hunter New England and the Lower Mid-North Coast area of New South Wales, Australia.

**Methods:**

A cross-sectional retrospective documentation survey was undertaken of case notes of young adults with type 1 diabetes accessing Hunter New England Local Health District public health services in 2010 and 2011, identified through ambulatory care clinic records, hospital attendances and other clinical records. Details of service usage, complications screening and evidence of vascular complications were extracted. Independent predictors were modelled using linear and logistic regression analyses.

**Results:**

A cohort of 707 patients were reviewed; mean (SD) age was 23.0 (3.7) years, with mean diabetes duration of 10.2 (5.8, range 0.2 - 28.3) years; 42.4% lived/ 23.1% accessed services in non-metropolitan areas.

Routine preventative service usage was low and unplanned contacts high; both deteriorated with increasing age. Low levels of complications screening were found. Where documented, hypertension, particularly, was common, affecting 48.4% across the study period. Diabetes duration was a strong predictor of vascular complications along with glycaemic control; hypertension was linked with renal dysfunction.

**Conclusion:**

Findings indicate a need to better understand young people’s drivers and achievements when accessing services, and how services can be reconfigured or delivered differently to better meet their needs and achieve better outcomes. Regular screening is required using current best practice guidelines as this affords the greatest chance for early complication detection, treatment initiation and secondary prevention.

## Background

Life expectancy and quality of life for people with type 1 diabetes are limited by the development of vascular complications such as nephropathy, retinopathy and hypertension, not least because such vascular changes lead to kidney failure and blindness, cardiac disease, stroke and limb amputations [1]. A systematic literature review of the prevalence and factors predictive of development of vascular complications in young adults with type 1 diabetes found few studies specifically recruited or even included representative samples, but that such complications were common even in youthful populations [2]. Few studies sought to determine factors predictive of development of these complications, but those most consistently reported, particularly amongst older groups, were diabetes duration, glycaemia and blood pressure (BP) control.

The onset of complications in early adulthood is particularly detrimental because of the loss of life years and restricted social participation at an age when it is more usually maximal. Early adulthood years are particularly important as this is when patterns of independent self-management are established. Young adults with type 1 diabetes may be particularly vulnerable to the development of complications as they may not receive disease-specific or age-appropriate care, and may disengage from the support of diabetes services when they leave the paediatric services they grew up with, at an age when adherence to diabetes self-management regimens is often reported as suboptimal [1,3-5]. Case note audit of young adults with type 1 diabetes accessing diabetes services in the state capital, a city and regional areas of New South Wales (NSW) in Australia demonstrated inadequate routine specialist care, poor self-management and frequent use of acute services for crisis management, particularly in regional areas [4]; however, sample sizes were small. Paucity of data on health service usage by young adults with type 1 diabetes for diabetes-related care, prevalence and predictors of vascular complications limits determination of the extent of the problem and hence appropriate service prioritisation.

This study aimed to identify the health service usage, prevalence and factors predictive of development of vascular complications (hypertension, retinopathy and nephropathy) in a cohort of young adults with type 1 diabetes in NSW.

## Methods

This cross-sectional retrospective documentation survey was part of the Youth Outreach for Diabetes (YOuR-Diabetes) project, an Australian National Health and Medical Research Council funded service development and evaluation initiative for young adults with type 1 diabetes in the Hunter New England (HNE) and Lower Mid-North Coast region of NSW. Research partners were the Australian Diabetes Council and Hunter New England Health (HNEH), the public health service provider for approximately 850,000 residents across 130,000 square kilometres of NSW, including metropolitan Newcastle and regional/rural areas [6]. Specialist services for type 1 diabetes in the region and more widely have been described elsewhere [4,7,8].

### Participants

Participants were young adults (aged 16–30 years) with type 1 diabetes as a primary condition. We collated our database from patient occasions of service with HNEH services from 2008 onwards, and audited contacts during 2010 and 2011. In Newcastle participants were identified through ambulatory care clinic records and Emergency Department (ED) and hospital attendances. In regional areas, records of Community Health, local diabetes educators and pathology services were also searched. We endeavoured to identify all young people with type 1 diabetes in the Local Health District, but recognised that our database may miss any who did not use state public health services, whose management and outcomes may be dissimilar to those reported.

### Data collection

Paper and electronic health records - individual case notes and multi-disciplinary documentation - were reviewed and data extracted using methods developed previously [4], and the criteria listed in Table 1; 95% agreement was demonstrated between two experienced data extractors.

**Table 1:** Study definitions of vascular complications

**Complication**	**Complication present when:**
**Hypertension**	Mean systolic or diastolic BP values ≥ 130/80 mmHg, respectively per annum, and/or prescription of anti-hypertensive medication
**Retinopathy**	Retinopathy documented
**Nephropathy**	At least one reported ACR measurement above laboratory threshold normal value

*ACR:* Albumin to creatinine ratio*. BP:* Blood pressure*.*

Planned and unplanned diabetes-related service contacts were the primary study outcomes: routine diabetes preventive care consultations with a doctor, diabetes nurse educator and/or dietitian; and unplanned presentations at any HNEH ED and/or acute hospitalisation for diabetes-related complaints. ED presentations resulting in hospital admission were solely recorded as hospitalisation.

Vascular complications were the secondary study outcomes. Data extracted included: BP measurements (number; values), ophthalmic examinations (number; documented absence/presence of retinopathy) and urinary albumin to creatinine ratio (ACR) measurements indicative of nephropathy (number; values): see Table 1 for definitions [9-11]. Data related to factors potentially predictive of development of vascular complications were also extracted. Socio-demographic data included age at diagnosis and area of residence [12] as these factors are recognized as impacting access and attendance at diabetes services [13,14].

Glycaemic control was determined by HbA1c assessments (number; values) classified in relation to an optimal target of less than or equal to 7.0% (53 mmol/mol) [9-11]. As the Australian Diabetes Society [15] advocates HbA1c be maintained at up to 8.0% (64 mmol/mol) for those with severe hypoglycaemic episodes or hypoglycaemia unawareness, mean HbA1c was further classified as equal to or above 8.0% (64 mmol/mol). Continuous Subcutaneous Insulin Infusion (CSII) use was noted as these devices have potential to improve diabetes management [16].

Data extracted for other factors shown to influence vascular disease risk were: smoking status, weight and height assessments, and Body Mass Index (BMI) calculations (number; values). BMI was firstly categorised as below 18.5 kg/m^2^ (underweight), 18.5 - 24.9 kg/m^2^ (on target), 25–29.9 kg/m^2^ (overweight) and equal to or above 30 kg/m^2^ (obese) [17].

Ethical approvals were obtained from Hunter New England, University of Newcastle and University of Technology, Sydney Human Research Ethics Committees.

### Analyses

Data were entered into SPSS version 21 for analyses. Frequencies, means and standard deviations were used descriptively according to the level of the variable for service usage, vascular complications and potential predictive factors. Relationships were examined between groups with and without vascular complications using Chi-square and t-tests; Pearson’s correlation coefficients were used between mean systolic and diastolic BP values and potentially linked characteristics such as planned, unplanned and total service contacts, mean HbA1c and BMI values, and diabetes duration. Associations between service usage, age and duration with diabetes were sought using multiple regression. Independent predictors of vascular complications were determined by logistic regression analysis, with separate models developed for hypertension, nephropathy and presence of any of the three vascular complications. For the analysis of these three models only, absence of evidence of documented vascular complications, laboratory values and smoking were treated as absence of that complication or potential predictive factor. No modelling was undertaken for retinopathy alone as reported cases were too few in number. Predictor variables were determined from the literature review and preliminary analyses of association. All three models included forced entry of the variables: any planned or unplanned health service contact, sex, metropolitan versus regional/rural residence, CSII use, smoking, mean HbA1c (used as a continuous variable and categorised as below, or equal to or above 8.0% (64 mmol/mol over the two years)), and diabetes duration [4,13,18-24]; hypertension was included as a variable in the analyses for nephropathy. BMI values were not included in regression analyses as assumptions of the analyses for a linear relationship were violated when used either as continuous or categorical variables. The critical level for retention in the model was set at 0.05. All assumptions of regression analysis were tested and met, including multi-collinearity.

## Results

A total of 707 individual case records were identified, with data available for 682 and 707 cohort members in 2010 and 2011, respectively. At the end of the two-year study period the mean (SD) age of the cohort was 23.0 (3.7) years. The sexes were approximately equally represented with 384 (54.3%) male; 39 (5.6%) were documented as Aboriginal and/or Torres Strait Islander. Mean (SD, range) diabetes duration was 10.2 (5.8, 0.2 - 28.3) years, with median 3.0 years of adult service usage. A minority of 299 (42.4%) cohort members lived and 112 (23.1%) accessed services outside of a major city. With no clear record of insulin delivery method for 103 (14.6%) cohort members, 154 (21.8%) were current and 36 (5.1%) intermittent CSII users. The profiles of CSII and non-CSII users differed: CSII users were significantly older (mean age 22.9 versus 21.5 years; t = 5.011, p < 0.001); had diabetes longer (mean 11.2 versus 9.8 years; t = 2.886, p < 0.004); received more planned service contacts/two years (mean 11.5 versus 6.25 contacts; t = 6.535, p < 0.001); more HbA1c measurements/ two years (mean 4.2 versus 2.6 measurements; t = 6.353, p < 0.001); more BP measurements/two years (mean 2.6 versus 1.5 measurements; t = 5.523, p < 0.001); and more ACR measurements/two years (mean 1.0 versus 0.7 measurements; t = 3.291, p < 0.002).

### Service usage

Routine health service usage was low; 280 (41.1%) and 306 (43.5%) cohort members had no planned service contact recorded during 2010 or 2011. Where a planned service did occur, a median of six individual planned contacts (range 1–52) with healthcare providers (i.e. consultations with doctors, nurses and dieticians) were undertaken across the two-year study period.

Unplanned service contacts were common; 308 (45.2%) and 326 (46.1%) members had at least one diabetes-related ED presentation and/or hospitalisation during 2010 or 2011; of those who had any unplanned contact an overall median of two contacts (range 1–22) occurred. Unplanned contacts occurred more frequently amongst those who had evidence of retinopathy or nephropathy: for example, 90% of those who had retinopathy versus only 61.4% of those without documented retinopathy had at least one unplanned service contact. A median of eight (range 1–62) planned/unplanned contacts were reported, with 178 and 184 (26.1% in each year) cohort members having no reported service contact, planned or unplanned, and 87 (12.8%) having no service contact over the two years.

There was a significant negative correlation between age and total number of planned contacts/two years (Pearson R = −0.339, p < 0.001) and significant but weaker association with duration since diagnosis (R = −0.168, p < 0.001; overall model fit R^2^ = 0.120). Multiple regression analysis demonstrated significant relationships between increasing age and fewer planned contacts (Beta = −0.321, p < 0.001), whilst the relationship with diabetes duration was not significant (overall model fit R^2^ = 0.118). A similar pattern was seen with unplanned service usage (Beta = −0.104, p < 0.019); whilst still significant, this was much weaker, i.e. increasing age was more strongly linked with reducing use of preventive care than acute service usage.

### Vascular complications

Low levels of screening and/or documentation were recorded but evidence indicated presence of co-morbid disease (Table 2). The majority had no documented BP measurement, ophthalmic examination or ACR measurement during either 2010 or 2011, respectively. Prescription records were unavailable for 269 (38%) and a prescription for anti-hypertensive medication was documented for 72 (10.2%). A total of 201 (48.4%) participants were classified as hypertensive on the basis of at least one documented elevated BP measurement or anti-hypertensive medication prescription. At least one documented BP measurement equal to or above 130/ 80 mmHg was reported in 35 (48.6%) cohort members prescribed anti-hypertensive medication, across the study period.

**Table 2:** Screening for vascular complications and associated outcomes

**Variable**	**2010**	**2011**
	***Number (%)***	***Number (%)***
**BP measurements documented**	(n = 682)	(n = 707)
At least one	313 (45.9)	306 (43.3)
**Mean systolic/diastolic BP**	(n = 313)	(n = 306)
≥ 130/80 mmHg	106 (33.9)	94 (30.7)
**Ophthalmic examinations documented**	(n = 682)	(n = 707)
At least one	95 (14)	85 (12)
**Ophthalmic examination reported outcome**	(n = 95)	(n = 85)
Retinopathy	13 (13.7)	8 (9.4)
**ACR measurements documented**	(n = 682)	(n = 707)
At least one	222 (32.6)	218 (30.8)
**ACR measurements above threshold value^**	(n = 219)	(n = 218)
At least one	33 (15.1)	35 (16.1)

*ACR:* Albumin to creatinine ratio*. BP:* Blood pressure*.*

*^* Three cohort members excluded from analysis for 2010 as measurement undertaken but result unknown*.*

Of those who had a documented ACR measurement, 137 (40.1%) had two or more and 17 (12.4%) of these had two or more above the threshold value. Those who used CSII were reported to have some form of vascular complication (hypertension, retinopathy and/or nephropathy) at a similarly high frequency to non-CSII users (55.6% affected versus 53.8%), but overall nearly 40% of the sample were eliminated from these analyses due to incomplete data (n = 428 included).

### Vascular risk factors

Low levels of documented risk factors were also evident (Table 3). Of those who had any HbA1c measurement a median of three measurements were documented (range 1–12) across the two year study period. In cohort members identified as having hypertension, retinopathy and/ or nephropathy, a minority of 67 (36.6%), 6 (30.0%) and 15 (26.8%) had mean recorded HbA1c below 8.0% (64 mmol/mol), respectively.

**Table 3:** Vascular disease risk factors

**Variable**	**2010**	**2011**
	***Number (%)***	***Number (%)***
**HbA1c documented**	(n = 682)	(n = 706)
At least one	422 (61.9)	425 (60.2)
**HbA1c value (s)** **≤** **7.0% (53 mmol/mol) documented in those with** **≥** **1 recorded**	(n = 422)	(n = 425)
At least one	104 (24.6)	95 (22.4)
**HbA1c value (s)** **≥** **8.0% (64 mmol/mol) documented in those with** **≥** **1 recorded**	(n = 422)	(n = 425)
At least one	295 (69.9)	293 (68.9)
**Mean HbA1c**	(n = 422)	(n = 425)
≥ 8.0% (64 mmol/mol)	260 (61.6)	269 (63.3)
**Weight documented**	(n = 683)	(n = 707)
At least one	340 (49.8)	345 (48.8)
**Mean BMI**	(n = 272)	(n = 255)
< 18.50 kg/m^2^	5 (1.8)	4 (1.6)
18.50 - 24.99 kg/m^2^	160 (58.8)	137 (53.7)
≥ 25–29.99 kg/m^2^	69 (25.4)	74 (29)
≥ 30 kg/m^2^	38 (14)	40 (15.7)

*BMI:* Body Mass Index

Although records of smoking were incomplete for 387 (54.7%), 94 (13.3%) were reported as current smokers. No weight was recorded for 38.8%, and a median one weight assessment per person (range 1–11) was documented. Height measurement was not documented for 259 (36.6%).

### Associations between risk factors and vascular complications

Cohort members were more likely to have documented hypertension if they were male (55.7% versus 41.0%, Chi^2^ = 9.02, p = 0.003) or had none rather than any unplanned service contact (55.4% versus 44.3%, Chi^2^ = 9.36, p < 0.003). Higher mean systolic BP values were linked to older age (r = 0.339, p < 0.001), longer diabetes duration (r = 0.168, p < 0.002), (unsurprisingly) higher mean diastolic values (r = 0.639, p < 0.001), greater BMI values (r = 0.210, p < 0.004), fewer planned (r = −0.167, p < 0.002), unplanned (r = −0.169, p < 0.001) and total service contacts (r = −0.201, p < 0.001).

Similar associations were seen with mean diastolic BP values, with higher recordings linked to older age (r = 0.302, p < 0.001), longer diabetes duration (r = 0.214, p < 0.001), greater mean HbA1c (r = 0.178, p < 0.001) and fewer planned (r = 0.109, p < 0.033) and total service contacts (r = −0.106, p < 0.038).

Cohort members who had retinopathy in comparison to those who did not were significantly older (mean age 24.1 versus 21.7 years, t = −3.053, df l58, p < 0.003), had longer diabetes duration (mean 14.1 versus 11.0 years; t = −2.531, df 143, p < 0.021) and more unplanned service contacts over the two-year period (mean 4.4 versus 1.8 contacts; t = 2.885, df 22, p < 0.009).

Cohort members who had any recorded ACR measurement above threshold values in comparison to those who did not were significantly older (mean 23.2 versus 22.0 years; t = −2.381, df 332, p < 0.018) and had significantly higher mean HbA1c values across the two-year period (9.4% versus 8.6%; t = −3.174, df 327, p < 0.002). They were more frequently documented with hypertension (68.2% versus 42.0%, Chi^2^ = 10.2, p < 0.001).

### Independent predictors of vascular complications

Logistic regression analysis revealed that cohort members were more likely to have hypertension (model Chi^2^ = 45.34, df 7, p < 0.001) if they had no (rather than any) health service contact (OR 0.21, 95% CI 0.1 - 0.51, p = 0.001), any use of CSII (OR 1.8, 95% CI 1.2 - 2.7, p = 0.004) or a longer diabetes duration (each year, OR 1.05, 95% CI 1.01 - 1.09, p = 0.006). The odds of having nephropathy (model Chi^2^ = 42.95, df 8, p < 0.001) were increased more than three times by having hypertension (OR 3.19, 95% CI 1.66 - 6.15, p < 0.001) and having a mean HbA1c at or greater than 8.0% (64 mmol/mol) (OR 3.59, 95% CI 1.67 - 7.74, p = 0.001) (Table 4).

**Table 4:** Predictors of hypertension, nephropathy and any vascular complication (hypertension, retinopathy and/or nephropathy)

**Predictor**	**Hypertension**			**Nephropathy**			**Hypertension, retinopathy and/or nephropathy**		
	***B***	**95% CI**	**p value**	***B***	**95% CI**	**p value**	***B***	**95% CI**	**p value**
**Any CSII use**	1.8	1.2 - 2.7	0.004	1.06	0.54 - 2.08	0.877	1.78	1.19 - 2.64	0.005
**Male**	1.42	0.97 - 2.08	0.069	0.58	0.28 - 1.03	0.062	1.16	0.8 - 1.68	0.424
**Mean HbA1c ≥ 8%**	1.42	0.96 - 2.09	0.077	3.59	1.67 - 7.74	0.001	1.64	1.13 - 2.38	0.01
**Smoking**	1.34	0.91 - 1.96	0.135	1.2	0.63 - 2.29	0.572	1.3	0.9 - 1.88	0.17
**Diabetes duration**	1.05	1.01 - 1.09	0.006	0.99	0.93 - 1.06	0.831	1.05	1.02 - 1.09	0.003
**Metropolitan residence**	0.92	0.63 - 1.37	0.69	1.184	0.6 - 2.32	0.623	0.97	0.66 - 1.42	0.868
**No reported service contact**	0.21	0.1 - 0.51	0.001				0.17	0.07 - 0.41	< 0.001
**Hypertension**	N/A			3.19	1.66 - 6.15	0.001	N/A		
**Constant**	0.16		< 0.001	0.3		< 0.001	0.21		< 0.001
**Model Statistics**	Chi^2^ = 45.34, df 7, n550, p < 0.001			Chi^2^ = 42.95, df 8, n550, p < 0.001			Chi^2^ = 58.02, df 7, n550, p < 0.001		

*CSII:* Continuous Subcutaneous Insulin Infusion*. N/A:* Not applicable*.*

No modelling undertaken for retinopathy independently or analyses of no health service contact as a predictor of nephropathy as too few cases.

The likelihood of documented hypertension, retinopathy and/or nephropathy (model Chi^2^ = 58.02, df 7, p < 0.001) increased with absence of health service contact (OR 0.17, 95% CI 0.07 - 0.41, p < 0.001), with any CSII use (OR 1.78, 95% CI 1.19 - 2.64, p = 0.005), a mean HbA1c equal to or above 8.0% (64 mmol/mol) (OR 1.64, 95% CI 1.13 - 2.38, p = 0.01) and longer diabetes duration (each year, OR 1.05, 95% CI 1.02 - 1.09, p = 0.003) (Table 4). Statistical significance was attenuated or lost for these variables when mean recorded values for HbA1c and BP were employed.

## Discussion

The study findings are broadly representative across metropolitan, regional and rural Australia as they present data from 707 young adults of a potential 830 people with type 1 diabetes within this age band registered on the National Diabetes Services Scheme (personal communication), a ‘best option’ source for population figures for this mobile group. Findings demonstrated young adults with type 1 diabetes in this region of NSW at risk of poor health outcomes. Low attendance for preventive care, and shortcomings according to international standards [9-11] in the screening they received when they attended, reduced sample size for many analyses; consequently some associations, whilst statistically significant, showed low explanatory power. Nonetheless, data indicated inadequate access/uptake of routine preventive care and increasing age of patients accompanied by a more significant pattern of reducing use of routine preventive care than use of acute services for diabetes crisis management.

Data were indicative or suggestive of co-morbid disease, consistent with systematic review findings [2]. Where assessed, one in six cohort members had at least one recorded episode of microalbuminuria and as many as one in three had a mean recorded systolic or diastolic BP equal to or above 130 mmHg and/or 80 mmHg, respectively; almost one in two were affected when medication for hypertension was included. One in nine had documented retinopathy; less than demonstrated for young adults in NSW between 1990–2000 [25], but consistent with more recent NSW adolescents’ data from 2005–2009 [26]. Whilst a reduction in retinopathy prevalence over time may have been related to changes in diabetes management following the definitive Diabetes Control and Complications Trial [22] which made glycaemic control central, low levels of screening potentially under-estimates the true level of retinopathy in this cohort; also hypertension and nephropathy. Collectively, these data are cause for concern, indicating low use of preventive services reducing with increasing age accompanied by early onset of co-morbid disease, increased risk of impaired quality of life and premature mortality.

Despite guidelines recommending use of angiotensin converting enzyme or angiotensin 2 receptor blockers even in children, few of this cohort were in receipt of treatment or treated to target. Only one in ten were documented as prescribed anti-hypertensive medication and of those that were, two in three had mean BP equal to or above 130/80 mmHg across the study period; potentially indicating missed opportunities for disease modification. This is particularly regrettable since elsewhere rates of anti-hypertensive prescription have been reported as increasing significantly, to 34.2% in 2007 [27].

Internationally accepted standards for glycaemia management were largely not met [9-11], with inadequate HbA1c monitoring and two-thirds of cohort members with at least one measurement within the two-year period having mean HbA1c at or greater than 8.0% (64 mmol/mol). However, the Epidemiology of Diabetes Interventions and Complications (EDIC) Study [28] found similar HbA1c values, increasing in adolescents in the intensive treatment group (from 8.1% to 8.4%) and decreasing (but still elevated) in the conventional treatment group (from 9.8% to 8.5%) after study end. These data suggested it may be difficult to maintain HbA1c values under 8.0% in young people outside a clinical trial. Data from this location in NSW appear consistent with this conclusion.

Findings regarding the use of CSII were noteworthy. Whilst CSII users received overall significantly greater planned service use and assessments, hypertension and any vascular complication occurred more frequently in association with any usage of CSII. Results should be viewed with caution due to missing data but whilst findings were not in line with some studies [16] they were consistent with a previous NSW study which showed that while CSII use doubled during the study period, HbA1c in users deteriorated, rising from 8.4% to 8.6% [3]. It is tempting to speculate that perhaps people with poor control of blood glucose, and associated hypertension, may have been started on CSII in an attempt to improve control, but most of these young people were likely to have commenced use of CSII as children. Education at initiation of CSII for children is primarily to parents/responsible adults. If the child/teen was not targeted for education pre-transition from paediatric care, deficiencies in CSII knowledge were not likely to have been picked up or rectified if the young adult did not have a good relationship with a pump-specialist. Subsidised access to CSII combined with greater access to specialist support for children has resulted in expansion of CSII use particularly amongst children [29], with trends to start CSII use at diagnosis gaining favour. This raises the question how prepared for independent self-management such CSII users are, as they move into adulthood and lose access to paediatric specialist services. Further research is needed to examine whether and how insulin pumps may deliver on their promise of improved diabetes control for people with type 1 diabetes [29]. Given the cost in provision of CSII and the human resources required to support pump users, the possibility that they may not improve outcomes is too important to ignore. Whilst there may be many reasons for a decline in management of diabetes and its complications in young adults using insulin pumps, one might be that stretched diabetes teams lack adequate specialist resources to provide the more complex and time consuming support needed to optimise results.

Over half of this cohort resided in a major city, and half of those who did not travelled there to access specialist diabetes services. Inadequate access to routine specialist care in regional areas may have contributed to low uptake and increasing attrition with age from routine preventive care services, infrequent screening and suboptimal outcomes. Geographical and socioeconomic factors have been cited as major issues in access to diabetes services, and strong predictors of attendance [14]. Improved diabetes management has been shown by those who maintain contact and relationships with their diabetes healthcare teams [13]. However, this may be a simplistic interpretation. Patterns of use of planned and unplanned service contacts suggest these young people were not routinely or systematically using preventive services to support their self-care; instead, emergency and acute services appeared to be being used with almost half of cohort members having at least one diabetes-related ED presentation and/or hospitalisation in 2010 and similarly in 2011. Emergency hospital admission can be seen as an indicator of poor quality of diabetes care [30], with concerns raised at the education provided by healthcare staff in this situation [31].

A chief limitation of this study was use of data originally collected as patient clinical healthcare records; all such studies are forced to rely on professional and legal accountability for clinical record-keeping, and the value attached to record quality in such situations of life-long care. Nonetheless study data will have been affected by factors affecting the quality of clinical record keeping. Whilst there was potential for Berkson’s bias on results, lack of access to General Practice (GP) or private practice data mean their service episodes were not reflected in these findings except as secondary report within case notes. However, few local GPs offered specialist support for type 1 diabetes; our previous qualitative study with this population reported their experience of GP diabetes care as predominantly age-inappropriate and non-specialist, and private endocrinologists as unaffordable [32]. Thus this may not have materially affected findings. The two-year time period of the study did not allow for trends across time, and the representative nature of these data can only be estimated by comparison to the earlier study, which revealed that little had changed over time [4]. Study co-morbidity definitions (specifically hypertension and nephropathy) were somewhat simplistic and data were not collected about acute illness and other co-morbidities that may affect screening and disease management. Furthermore, considering the high prevalence of type 2 diabetes in Aboriginal and/or Torres Strait Islander populations [33,34] a few may have been documented incorrectly with type 1 diabetes based on insulin administration. Nonetheless, the strengths of this study lie with the size of the cohort, the geographical size and range from which the cohort derives, and its near-complete population sampling within this under-researched age group.

## Conclusion

Findings flag a need to better understand young people’s drivers and achievements when accessing services, and how services can be reconfigured or delivered differently to engage young people with age-appropriate care that better meets their needs to achieve improved outcomes and defer development of complications. In line with national guidelines [10], most type 1 diabetes should be managed by a multidisciplinary specialist health-care professional team, including in the rural and regional setting, where diabetes care may be provided by a locally based paediatrician, physician and/or GP on a shared care basis with a multidisciplinary diabetes care team. In many locations this will require reconfiguration and appropriate apportionment of resourcing of multidisciplinary teams in both urban and rural areas, particularly in view of the highly specialist needs of the increasing number of people with diabetes using CSII. Health professionals need to work out ways to enable regular screening to be performed using current best practice guidelines as this affords the greatest chance for early complication detection and hence for initiation of treatment and secondary prevention.

## Competing interests

The authors declare that they have no competing interests.

## Author’s contributions

Study proposal developed by LP, JL, JD, PM, SA and KS. Data collected by JD. Analyses conducted by SJ, LP, RG and PM. Paper drafted by SJ, LP, RG and JL, revised and agreed by all authors. All authors read and approved the final manuscript.

## Author’s information

SJ: RN, CDE Doctoral student, Faculty of Health, University of Technology, Sydney, Australia.

LP: PhD, MSc, RN Professor of Nursing Research and Practice Development, Prince of Wales Hospital, Sydney and Sydney Eye Hospitals, Faculty of Health, University of Technology, Sydney, Australia.

RG: PhD, MN, RN Associate Professor Chronic and Complex Care, and Director, Research Students, Faculty of Health, University of Technology, Sydney, Australia.

JL: MBChB, MRCP, FRCP, MMedSci Associate Professor, University of Toronto, Canada.

JD: PhD, BAg Research Assistant, University of Newcastle, Australia.

PM: PhD, BMath, Grad Dip Med Stats Associate Professor, University of Newcastle, Australia.

SA: MBBS, FRACP Staff Specialist Endocrinologist, Lead Clinician, Diabetes Service, Hunter New England Local Health District, Australia.

KS: MBBS, FRACP, PhD Medical Foundation Chair in Adolescent Medicine, University of Sydney, Australia.

## Pre-publication history

The pre-publication history for this paper can be accessed here:

http://www.biomedcentral.com/1472-6823/14/39/prepub
